# Proteomics and Co-expression Network Analysis Reveal the Importance of Hub Proteins and Metabolic Pathways in Nicotine Synthesis and Accumulation in Tobacco (*Nicotiana tabacum* L.)

**DOI:** 10.3389/fpls.2022.860455

**Published:** 2022-04-28

**Authors:** Zejun Mo, Lili Duan, Yuanyuan Pu, Zonglin Tian, Yuzhou Ke, Wen Luo, Kai Pi, Ying Huang, Qiong Nie, Renxiang Liu

**Affiliations:** ^1^College of Agriculture, Guizhou University, Guiyang, China; ^2^Key Laboratory of Tobacco Quality in Guizhou Province, Guiyang, China; ^3^College of Tobacco, Guizhou University, Guiyang, China

**Keywords:** nicotine, proteomics, weighted gene co-expression network analysis, metabolites, enzyme activity, *Nicotiana tabacum*

## Abstract

Nicotine is a unique alkaloid present in tobacco that is widely used in cigarettes and in the agricultural, chemical, and pharmaceutical industries. However, the research on nicotine is mostly limited to its synthesis pathways, and only a few studies have explored the effects of other metabolic pathways on nicotine precursors. Regulating the nicotine content in tobacco can greatly promoting the application of nicotine in other fields. In this study, we performed global data-independent acquisition proteomics analysis of four tobacco varieties. Of the four varieties, one had high nicotine content and three had a low nicotine content. A total of 31,259 distinct peptides and 6,018 proteins across two samples were identified. A total of 45 differentially expressed proteins (DEPs) co-existed in the three comparison groups and were mainly involved in the transport and metallic processes of the substances. Most DEPs were enriched in the biosynthesis of secondary metals, glutathione metabolism, carbon metabolism, and glycolysis/gluconeogenesis. In addition, the weighted gene co-expression network analysis identified an expression module closely related to the nicotine content (Brown, *r* = 0.74, *P* = 0.006). Gene Ontology annotation and Kyoto Encyclopaedia of Genes and Genomes enrichment analysis showed that the module proteins were mainly involved in the synthesis and metabolism of nicotine precursors such as arginine, ornithine aspartate, proline, and glutathione. The increased levels of these precursors lead to the synthesis and accumulation of nicotine in plants. More importantly, these proteins regulate nicotine synthesis by affecting the formation of putrescine, which is the core intermediate product in nicotine anabolism. Our results provide a reference for tobacco variety selection with a suitable nicotine content and regulation of the nicotine content. Additionally, the results highlight the importance of other precursor metabolism in nicotine synthesis.

## Introduction

Plants produce various secondary metabolites that are vital for growth and environmental adaptation (Sanchez and Demain, 2019). Alkaloids are one of the most important secondary metabolites that play an important role in defence against herbivores and insects (Baldwin, 1998; Steppuhn et al., 2004). Nicotine is a major alkaloid that is produced specifically in tobacco, accounting for approximately 90–95% of total alkaloids and 0.6–3.0% of leaf dry weight (Hibi et al., 1994; Sanchez and Demain, 2019). Nicotine has an extremely high utilisation value in various industries (Mukhopadhyay et al., 2004; Tang et al., 2007), such as medicine (Cloëz-Tayarani and Changeux, 2007; Hao et al., 2017), and agriculture (Thurston et al., 1966; Cloëz-Tayarani and Changeux, 2007; Hao et al., 2017; Lewis et al., 2020). Nicotine is used for the commercial production of tobacco, and the nicotine content directly affects the inherent quality, safety, and availability of cigarette products (Nölke et al., 2018). The World Health Organisation has recommended reducing the nicotine content in cigarettes to reduce overall addiction to such products (World Health Organization, 2009). Therefore, monitoring the nicotine content of tobacco is essential for the tobacco industry and multi-purpose utilisation of nicotine.

Nicotine is synthesised only in the roots of tobacco and is then translocated to the aerial parts of the plant *via* the xylem and finally accumulates in the leaves (Dewey and Xie, 2013; Shoji and Hashimoto, 2013). These processes are affected by various external factors and internal genetic factors such as plant variety (Tian et al., 2018), climate (Jung et al., 2009; Zhang et al., 2019), soil environment, and cultivation measures (Baldwin, 1989; Cane et al., 2005). Studies have suggested that the tobacco nicotine content is regulated by many factors; however, the ability of nicotine accumulation is mainly determined by the genetic characteristics of varieties (Legg et al., 1969; Dewey and Xie, 2013). Thus, the most effective and preferred method for obtaining new materials with an appropriate nicotine content is the application of modified plant genetics, which involves studying nicotine-related genes and their transcription and translation products (Lewis, 2018).

The biosynthesis of nicotine involves the formation of a pyridine ring and a pyrrole ring, as well as their combination (Baldwin, 1999). This process is mediated by many key enzymes, transcription factors, and associated genes. Several studies have shown that ornithine decarboxylase and arginine decarboxylase provide putrescine for the synthesis of nicotine (Marton and Pegg, 1995; Imanishi et al., 1998; Tiburcio and Galston, 2016). The putrescine *N*-methyltransferase (PMT) is a rate-limiting enzyme involved in the formation of the pyrrole ring in nicotine biosynthesis, which affects the flow rate of putrescine (Chou and Kutchan, 1998). PMT overexpression can increase the nicotine content in leaves (Sato et al., 2011). The transcription level of the *N*-methylputrescine oxidase (MPO) gene is also the key for restricting nicotine synthesis (Wagner et al., 1986). The gene catalyses the conversion of N-methylputrescine into 4-methylaminobutyl ether (Chou and Kutchan, 1998). Wagner et al. (1986) showed that quinolinic acid phosphoribosyl transferase (QPT) is another rate-limiting enzyme involved in nicotine synthesis; the stronger the QPT activity in root tissues, the higher is the nicotine content of the tobacco plant. NtMYB305a regulates the expression of multiple genes associated with the nicotine metabolism pathway including the PMT gene; regulates nicotine synthesis; and cooperates with the G-box binding protein NtMYC2a to regulate nicotine metabolism in tobacco (Bian et al., 2022). In addition, the inhibition of MYCla and MYClb expressions was reported to decrease the nicotine content in tobacco B-Y-2 cells, indicating that MYCla and MYClb are the positive regulators of nicotine synthesis (Zhang et al., 2012). The discovery of these key factors is of great significance for the directional regulation of nicotine synthesis and accumulation.

To date, many studies have explored the molecular mechanisms by using “omics” methods such as transcriptomics (Lou et al., 2017; Yuan et al., 2021), proteomics (Hu et al., 2017; Hochholdinger et al., 2018), and metabolomics (Kumar et al., 2017; Sharma et al., 2021). By comparing the omics expression characteristics between different samples, the genes controlling various phenotypes can be identified (Jung et al., 2021). Previous studies have mostly investigated the effect of a certain gene or enzyme in the nicotine synthesis pathway; however, none of the studies have investigated the key regulatory factors affecting nicotine synthesis and metabolism from the perspective of proteomics (Wang et al., 2011; Yin et al., 2018). In this study, we performed a comprehensive proteomics analysis of root tips with different nicotine contents. The results of this study provide a basis and reference for obtaining new tobacco varieties with a suitable nicotine content.

## Materials and Methods

### Plant Material and Analysis of Nicotine Traits

**FIGURE 1:**
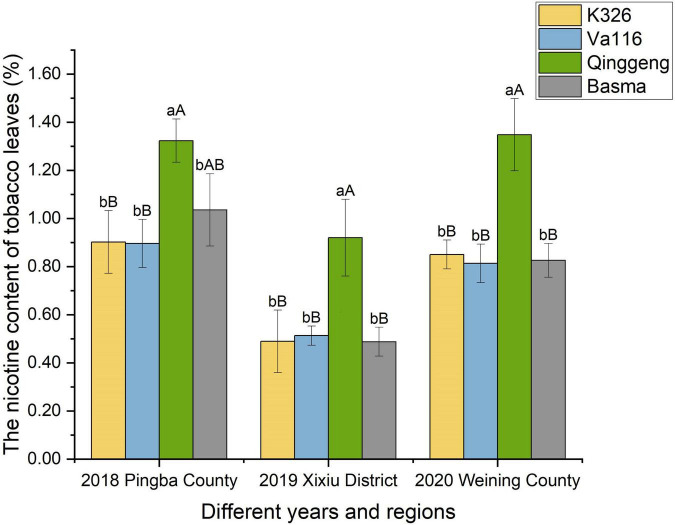
The difference in the nicotine content among four tobacco varieties from different regions (Pingba County, Xixiu District, and Weining County) and years (2018, 2019, and 2020). The average of three biological replicates per material was used for mapping. Error bars represent significant difference of three replicates. Significant differences among the nicotine contents at *P* < 0.05 and *P* < 0.01 were determined using the Duncan’s new multiple range test. The lowercase alphabets represent a significant difference (*P* < 0.05); uppercase alphabets represent an extremely significant difference (*P* < 0.01).

Through many years of repeated experiments, four tobacco varieties were screened out to elucidate the mechanism of nicotine synthesis and accumulation. All varieties were provided by the Institute of Tobacco, the Chinese Academy of Agricultural Sciences. One variety was sun-cured tobacco (Qinggeng) with a high nicotine content, and the other three varieties (Va116, Basma, and K326) were flue-cured tobacco with a low nicotine content (Supplementary Table 1 and Figure 1).

Using a randomised block design, a field experiment was performed at the tobacco research experimental base of Guizhou University in 2018 (Pingba County), 2019 (Xixiu District), and 2020 (Weining County), respectively. The experiment was performed in triplicate. The intra- and inter-row spacing of plants was 110 and 55 cm. All plants were topped on the same day when more than 50% of the plants flowered (68th day after transplanting). Samples were collected on day 75 after transplantation. Refer to Mo et al. (2021b) for the collection method of experimental samples. The root tip samples were evenly mixed in equal quantities, washed with clear water, and rinsed with phosphate-buffered saline. Then, the samples were kept in different sterilised centrifugation tubes frozen with liquid nitrogen and immediately stored in a refrigerator at −80°C. These root samples were used for subsequent proteomics analysis, enzyme activity measurement. The enzymes in the leaf samples were denatured at 105°C for 30 min, after which the samples were dried at 75°C, ground into powder, bagged, and sealed for storage. According to the method of Shoji et al. (2009), nicotine was separated from the extract of dry leaf samples and analysed through gas chromatography.

### Protein Extraction and Mass Spectrometry

The protein samples were prepared using the trichloroacetic acid-acetone precipitation method. The protein concentration was determined using a BCA protein concentration determination kit according to the manufacturer’s instructions (Source: Bio-rad, Specifications and models: 5000201).

### Protein Enzymolysis and Mass Spectrometry

Protein enzymolysis was performed according to the method reported by Li et al. (2019) and Xu (2019). Other parameters and operation methods were performed according to a study by Mo et al. (2021b). Mass spectrometry was performed according to the methods described by Li et al. (2017), Cui (2018), and Dong et al. (2019).

### Processing and Analysis of Proteomics Data

Data Dependent Acquisition (DDA) data were searched using the programme Maxquant (Maxquant_1.5.3.17). The database was downloaded from tobacco_uniprot, and the iRT peptide fragment sequence was added to the database. The parameters were set according to the protocols described by Fu et al. (2019) and Dong et al. (2019). The original raw files and the search results were exported to the software Spectronaut (Spectronaut Pulsar Xerox 12.0.20491.4) to construct a spectral library. The following software parameters were set: retention time prediction type was set to dynamic iRT; interference on MS2 level correction was enabled; and cross run normalisation was enabled. All the results were filtered by setting a parameter Q value cut-off of 0.01 (equivalent to the false discovery rate <1%).

### Bioinformatics Analyses

In order to monitor and evaluate the stability of the system and the reliability of the experimental data, one quality control (QC) sample (generally a mixed sample mix of all samples) was inserted in the sample cohort, and the data consistency of the QC samples inserted throughout the experiment was evaluated. Within-group Pearson correlation analysis was mainly used to assess the QC. The closer the correlation coefficient is to 1, the more stable the experimental system is quality control. A hierarchical clustering algorithm was used to analyse differentially expressed proteins (DEPs) among the compared groups. The package Complex Heatmap R (R Version 3.4) was used to classify the two dimensions of samples and protein expression simultaneously (distance algorithm: Euclid, connection mode: Average linkage) and to generate a hierarchical clustering heat map (Mo et al., 2021b). The Gene Ontology (GO) function of the identified protein was annotated using the software Blast2Go^1^ (Conesa et al., 2005). The Cluster of Orthologous Groups of proteins (COG) analysis was performed using the OmicShare tools.^2^ The Fisher’s exact test was used for the GO functional enrichment analysis of DEPs (Liu et al., 2021). The Kyoto Encyclopaedia of Genes and Genomes (KEGG)^3^ pathway of the target protein set was annotated using the KEGG Automatic Annotation Server (KAAS) (Moriya et al., 2007; Kanehisa et al., 2012).

### Weighted Gene Co-expression Network Analysis

The weighted gene co-expression network analysis (WGCNA) algorithm is commonly used for constructing a gene co-expression network. In the gene co-expression networks, genes with common expression in different samples are present in the same gene network, and the co-expression relationship between the genes is generally determined by the expression correlation coefficient between those genes (Duan et al., 2020). A commonly expressed gene module was obtained and then connected with the concerned phenotypic information to determine the relationship between the gene network and the phenotype, as well as the core genes in the network. WGCNA was performed using the R language package (Langfelder and Horvath, 2008).

### Determination of Enzyme Activity

Enzyme activity was determined using a double-antibody one-step sandwich enzyme-linked immunosorbent assay, according to the specific operation methods described by Pu (2020).

### Statistical Analyses

Duncan’s new multiple range test was used to analyse variations in nicotine contents among the groups (*P* < 0.05) by using SPSS version 16.0.

## Results

### Analysis of the Nicotinic Content in Different Genotypes

To understand the differences in nicotine synthesis and accumulation capacity among the four genotypic materials, we determined the nicotine content of tobacco leaves through gas chromatography. The nicotine content of Qinggeng was found to be significantly higher than those of K326, Basma, and Va116; however, no significant difference was observed in the nicotine content between the three varieties K326, Basma, and Va116 (Figure 1). Simultaneously, the difference between the nicotine content of different varieties was consistent at different time points and regions. These results indicated that the nicotine content of the four varieties exhibit real and stable differences at the genetic level.

### Inventory of Root Proteins Identified by Data-Independent Acquisition

**FIGURE 2:**
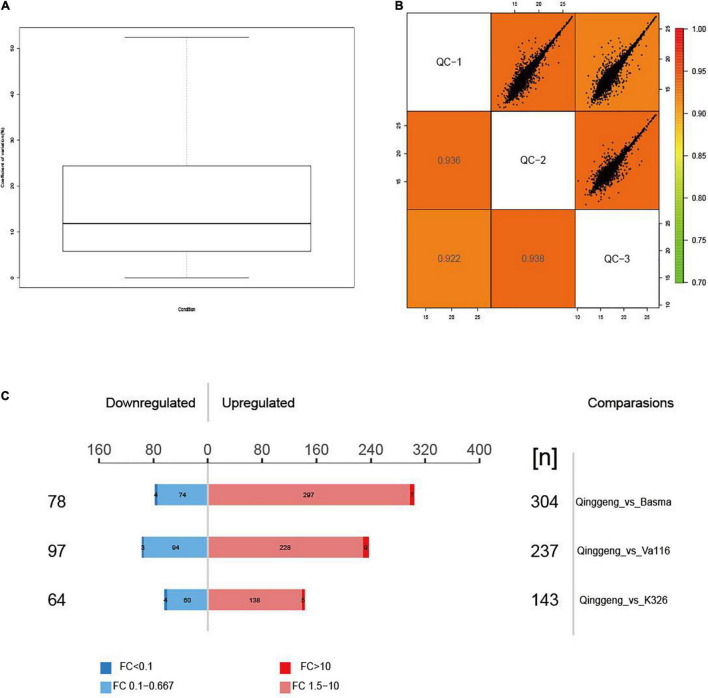
The protein expression abundance test of four tobacco varieties. **(A)** The distribution of coefficient of variation (CV) of quality control samples. **(B)** The correlation analysis of quality control samples. **(C)** Display of differentially expressed proteins in the three comparison groups.

To elucidate the mechanisms of different nicotine contents, we performed a data-independent acquisition (DIA) proteomics analysis of roots in the maturation stage. By using the protein analysis systems, we identified 31,259 distinct peptides and 6,018 proteins across all twelve samples. The detailed information of the identified peptides and proteins is presented Supplementary Files 1, 2. The coefficient of variation (CV) and Pearson’s correlation coefficient analysis showed that the whole experimental system was stable and that the data were reliable (Figures 2A,B). A total of 3,850 proteins were found to co-exist in all four varieties. We identified 42 proteins only in Qinggeng and 570 proteins co-existing in the other three varieties (Figure 2C).

**FIGURE 3:**
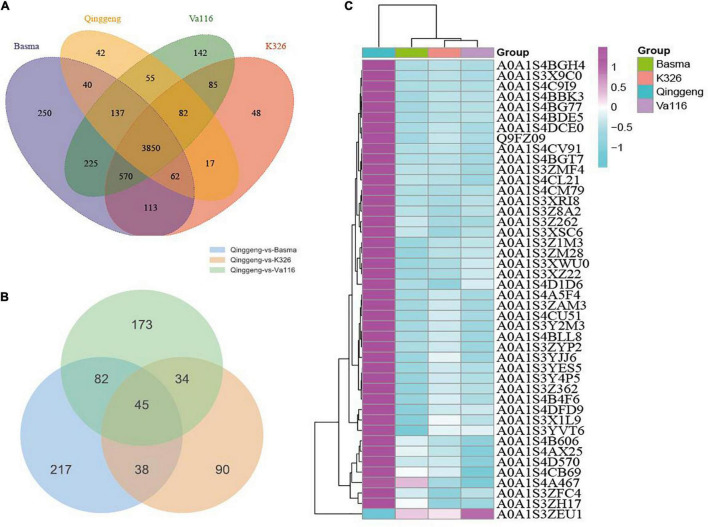
The differentially expressed protein (DEP) profiles. **(A)** Venn diagram of DEPs between the four tobacco varieties. **(B)** Venn diagram of DEPs in the comparison groups Qinggeng vs. Basma, Qinggeng vs. Va116 and Qinggeng vs. K326. **(C)** Hierarchical cluster analysis of DEPs among the varieties.

Proteins with the threshold criteria (*P*-values < 0.05 and up fold change >1.5 or down fold change <0.67) were considered as DEPs and identified through pairwise comparisons. We identified a total of 382 DEPs, including 304 upregulated and 78 downregulated proteins, by comparing Qinggeng with Basma. Furthermore, by comparing Qinggeng with Va116, we obtained 334 DEPs, including 237 upregulated and 97 downregulated proteins. Moreover, we observed 207 DEPs, including 143 upregulated and 64 downregulated proteins, by comparing Qinggeng with K326 (Figure 3A). The Venn diagram analysis revealed that 45 DEPs (44 increased and 1 decreased in abundance) were common in the three comparisons (Table 1 and Figure 3B). Subsequently, we performed a hierarchical clustering analysis of these 45 DEPs, and the results indicated that the protein expression patterns in Qinggeng were contrary to those in K326, Basma, and Va116 (Figure 3C). We speculate that these specifically expressed proteins might be involved in nicotine synthesis and accumulation processes.

**TABLE 1:** Verification of 45 candidate proteins relating to nicotinic content by data-independent acquisition (DIA) proteomics.

Protein accessions	Qinggeng-vs-K326		Qinggeng-vs-Va116		Qinggeng-vs-Basma	
			
	Foldchange	*P*-value	Foldchange	*P*-value	Foldchange	*P*-value
A0A077D9R7	2.2372	0.0314	3.6675	0.016	3.8662	0.0073
A0A1S3X1L9	1.8154	0.0127	2.2871	0.0054	3.3453	0.0006
A0A1S3X9C0	1.9254	0.0433	2.0064	0.0119	2.0135	0.0163
A0A1S3XRI8	12.3364	0.0035	7.6067	0.0007	9.5147	0.0003
A0A1S3XSC6	2.749	0.017	2.3725	0.0221	2.1672	0.0153
A0A1S3XWU0	3.8989	0.005	2.9235	0.0016	5.1317	0.0072
A0A1S3XZ22	2.1936	0.0326	1.8756	0.0371	2.299	0.0269
A0A1S3Y2M3	1.923	0.022	2.3633	0.0363	2.2814	0.006
A0A1S3Y4P5	1.7103	0.0432	1.8677	0.0402	2.0477	0.0289
A0A1S3YES5	2.0235	0.0058	2.2907	0.0082	2.5996	0.0018
A0A1S3YJJ6	1.5505	0.0212	1.8831	0.0342	1.9288	0.0059
A0A1S3YVT6	1.74	0.0376	1.7479	0.0407	3.1845	0.0082
A0A1S3Z1M3	2.5906	0.03	2.4278	0.0001	3.1861	0.0011
A0A1S3Z262	4.9199	0.0025	4.7954	0.0067	3.248	0.0058
A0A1S3Z362	2.506	0.0337	2.9902	0.0081	4.0941	0.0061
A0A1S3Z8A2	2.0497	0.0338	1.8947	0.0199	1.8853	0.0357
A0A1S3ZAM3	2.6938	0.0116	3.9107	0.0009	3.9052	0.0007
A0A1S3ZEU1	0.4366	0.0484	0.3332	0.0029	0.4135	0.0005
A0A1S3ZFC4	3.75	0.0036	2.6222	0.031	2.2547	0.0193
A0A1S3ZH17	3.1532	0.0213	2.9413	0.004	1.858	0.0266
A0A1S3ZM28	3.5936	0.0157	3.2592	0.0096	5.8153	0.0008
A0A1S3ZMF4	2.5843	0.0282	3.0901	0.0142	2.6267	0.0021
A0A1S3ZYP2	3.0193	0.0173	5.5358	0.0114	4.2152	0.02
A0A1S4A467	3.7814	0.0026	11.2858	0.0025	1.5461	0.0236
A0A1S4A5F4	2.5469	0.0257	3.3069	0.0196	3.3226	0.0031
A0A1S4AX25	2.0854	0.0182	3.0254	0.0254	1.7959	0.0045
A0A1S4B4F6	1.8778	0.046	2.2252	0.0144	2.4889	0.0388
A0A1S4B606	2.8878	0.0006	4.1674	0.0002	2.2391	0.023
A0A1S4BBK3	3.0738	0.0043	3.3011	0.0026	3.8775	0.0012
A0A1S4BDE5	2.2963	0.0284	2.2334	0.022	2.4653	0.0255
A0A1S4BG77	5.5468	0.0007	5.663	0.0011	8.8572	0.0005
A0A1S4BGH4	3.828	0.0183	4.3561	0.0177	4.3471	0.0039
A0A1S4BGT7	4.1607	0.0112	5.5006	0.0059	4.5187	0.0047
A0A1S4BLL8	1.8782	0.0226	2.3093	0.0173	2.1986	0.0177
A0A1S4C9I9	12.0719	0.0003	15.5064	0.0003	18.6282	0
A0A1S4CB69	2.2688	0.0093	7.5137	0.0099	2.0098	0.0162
A0A1S4CL21	2.8087	0.0073	3.3608	0.0092	2.6423	0.0097
A0A1S4CM79	8.112	0.0092	7.2671	0.0015	7.2056	0.0011
A0A1S4CU51	3.6026	0.0366	7.0352	0.0102	8.62	0.0096
A0A1S4CV91	3.7617	0.0285	5.1916	0.0287	4.2947	0.0291
A0A1S4D1D6	3.8491	0.0036	2.3257	0.0047	2.9955	0.0035
A0A1S4D570	1.6792	0.0331	2.159	0.023	1.6921	0.0396
A0A1S4DCE0	4.0947	0.0195	5.7703	0.0093	5.6024	0.028
A0A1S4DFD9	1.5846	0.0108	1.585	0.0046	1.9595	0.007
Q9FZ09	2.5305	0.0449	2.9379	0.0058	3.1819	0.0052

### Classification of Differentially Expressed Proteins Identified Through Data-Independent Acquisition

**FIGURE 4:**
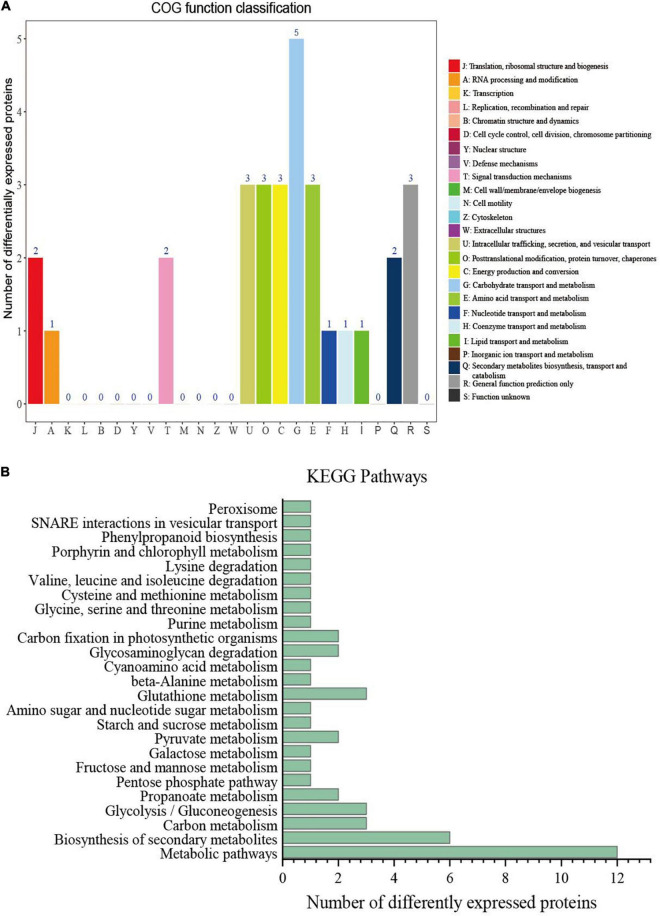
Clusters of orthologous groups (COG) functional annotation and Kyoto Encyclopedia of Genes and Genomes (KEGG) enrichment analysis of differentially expressed protein (DEPs). **(A)** COG function classification of 45 co-expressed DEPs. **(B)** KEGG function classification of 45 co-expressed DEPs.

To investigate the biological function of these DEPs, we classified the identified DEPs into 13 categories by using the Clusters of Orthologous Groups (COG) database. The largest category was carbohydrate transport and metabolism (5 DEPs), followed by intracellular trafficking, secretion, and vesicular transport (3 DEPs), post-translational modification, protein turnover, and chaperones (3 DEPs); energy production and conversion (3 DEPs); amino acid transport and metabolism (3 DEPs); and general function prediction only (3 DEPs) (Figure 4A). The classification showed that many co-expressed differential proteins were involved in the transport and metabolic processes of the substances. Proteins with these functions may play an important role in the transport of nicotine, so as to promote the transfer and accumulation of nicotine synthesised by roots to leaves.

To understand the functional consequences of the DEPs associated with the nicotine content, we performed KEGG pathway mapping. The annotation results were divided into 11 categories, mainly involving global and overview maps categories, followed by carbohydrate metabolism and amino acid metabolism (Figure 4B). The KEGG terms such as biosynthesis of secondary metabolites, glutathione metabolism, carbon metabolism, and glycolysis/gluconeogenesis were highly enriched in the co-expressed DEPs. Some of the terms were found to be related to the metabolic processes of nicotine such as amino sugar and nucleotide sugar metabolism and phenylpropanoid biosynthesis. The results suggested that these proteins from different physiological and metabolic pathways jointly regulate nicotine accumulation.

### Visualisation of the Nicotine Content Hub Protein

**FIGURE 5:**
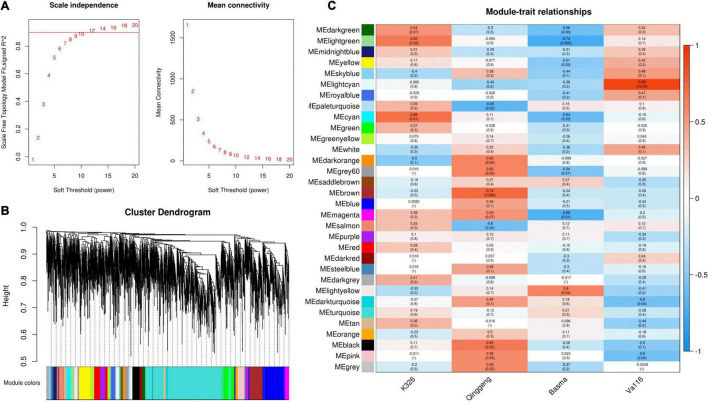
Weighted gene co-expression network analysis of differentially expressed protein (DEPs) in the four tobacco varieties. **(A)** Determination of soft threshold (power). Left: Scale-free topology fit index as a function of different soft threshold (power), the line represents that R2 = 0.9; Right: Mean connectivity as a function of different soft threshold (power). **(B)** Clustering dendrograms of protein and module division, with dissimilarity based on the topological overlap, together with assigned module colours. Overall, 32 co-expression modules were constructed and are shown in different colours. These modules ranged from large to small according to the number of genes included. **(C)** Module-sample group association analysis. Each row corresponds to a module, labelled with colour as in panel **(B)**, and each column corresponds to a sample group. The colour of each cell at the row-column intersection indicates the correlation coefficient between the module and the sample group.

Weighted gene co-expression network analysis (WGCNA) is a common systematic network analysis method. Using WGCNA to analyze transcriptomic and genomic data, we can identify trait related gene modules and hub genes and find biomarkers (Lu et al., 2019). To further analyse changes in proteins during nicotine synthesis and identify the hub proteins that affect the nicotine content, we finally selected 4,594 proteins from root tip samples to construct a co-expression module by using the WGCNA package tool. The power Estimate function in the WGCNA software package was used to estimate the optimal power value (Supplementary File 3). When the power value was 12, the independence degree was up to 0.9 and the average connectivity tended to be 0 (Figure 5A). The results showed that the power value provided a scale-free network that met the requirements and contained sufficient information. Therefore, the cluster analysis was performed on the network topology overlap matrix calculated with a power value of 12. Finally, 32 co-expression modules were identified, with different colours representing different modules (Figure 5B). The results of correlation analysis between each protein co-expression module and nicotine content are shown in Figure 5C. The brown module exhibited a significant positive correlation with the nicotine content (*r* = 0.74, *P* = 0.006), which could be used as a key module for subsequent analysis.

**FIGURE 6:**
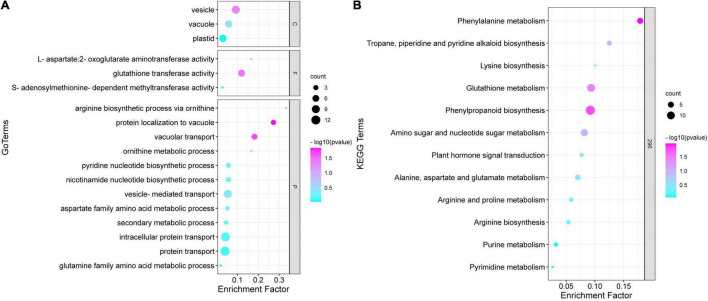
Gene Ontology (GO) functional annotation and Kyoto Encyclopedia of Genes and Genomes (KEGG) enrichment analysis of brown module proteins. **(A)** Gene Ontology function classification of the brown module. **(B)** Kyoto Encyclopaedia of Genes and Genomes function classification of the brown module.

The GO annotation of the brown module indicated that the biological processes involved in proteins mainly include two aspects, namely transport and metabolism (Figure 6A). The transport processes included protein transport, intracellular protein transport, and vesicle transport, whereas the metabolic processes included the ornithine metabolic process, aspartate family amino acid metabolic process, and secondary metabolic process. In terms of cellular components, proteins were mainly associated with the plastid, vacuole, and vesicle composition. Moreover, some proteins were associated with the molecular functions such as enzyme activity. The KEGG analysis for the critical modules indicated that the major mapped pathways included those related to the biosynthesis of other secondary metabolites and amino acid metabolism, such as alanine, aspartate, and glutamate metabolism; arginine and proline metabolism; and glutathione metabolism (Figure 6B). According to these important GO terms and metabolic pathway, some hub proteins were extracted (Table 2). More importantly, most of these pathways directly or indirectly affected the biosynthesis of putrescine and then regulated the synthesis and transport of nicotine in tobacco roots. The results showed that putrescine was the core intermediate product in nicotine anabolism.

**TABLE 2:** Description information of the key proteins of nicotine synthesis.

Protein accession	Protein description	Gene accession	Symbol	Enzyme number
A0A1S3ZW98	Amine oxidase	LOC107791110	AOC3, AOC2, tynA	EC:1.4.3.21
A0A1S3ZLW1	Aspartate aminotransferase, mitochondrial	LOC107788146	GOT2, AAT	EC:2.6.1.1
A0A1S4CP92	Gamma aminobutyrate transaminase 3, chloroplastic isoform X1	LOC107821173	POP2	EC:2.6.1.96
A0A1S4DRP0	Gamma aminobutyrate transaminase 3, chloroplastic-like isoform X1	LOC107832742	POP2	EC:2.6.1.96
A0A1S3Z7A0	Argininosuccinate lyase, chloroplastic	LOC107783840	argH, ASL	EC:4.3.2.1
A0A1S3XTH4	Basic form of pathogenesis-related protein 1-like	LOC107768378	PR1	
A0A1S3Y9V6	TGACG-sequence-specific DNA-binding protein TGA-2.1-like	LOC107773895	TGA	
A0A1S3XCC7	LL-diaminopimelate aminotransferase, chloroplastic-like	LOC107763425		EC:2.6.1.83
A0A1S4AYR2	Aldehyde dehydrogenase family three member H1-like	LOC107802760	ALDH	EC:1.2.1.3
A0A1S3Z7A0	Argininosuccinate lyase, chloroplastic	LOC107783840	argH, ASL	EC:4.3.2.1
A0A1S3YES5	Inosine-5’-monophosphate dehydrogenase	LOC107775294	IMPDH, guaB	EC:1.1.1.205
A0A1S4DKL8	Pyruvate kinase	LOC107830802	PK, pyk	EC:2.7.1.40
A0A1S4DK48	UMP-CMP kinase	LOC107830661	CMPK1, UMPK	EC:2.7.4.14
A0A1S4C3N5	Puromycin-sensitive aminopeptidase-like 1	LOC107814831	CARP, pepA	EC:3.4.11.1
A0A1S3Z195	5-oxoprolinase-like	LOC107781908	OPLAH, OXP1, oplAH	EC:3.5.2.9
A0A1S3Z262	Puromycin-sensitive aminopeptidase-like 1	LOC107782212	ANPEP, CD13, pepN	EC:3.4.11.2
A0A1S3XWK1	Glutathione S-transferase U17-like	LOC107769388	GST, gst	EC:2.5.1.18
A0A1S4BDE5	Probable glutathione S-transferase	LOC107807101	GST, gst	EC:2.5.1.18
A0A1S3Y2B9	Glutathione S-transferase U9-like	LOC107771507	GST, gst	EC:2.5.1.18
A0A1S3YEE6	Glutathione S-transferase U9-like	LOC107775362	GST, gst	EC:2.5.1.18
A0A1S4D1D6	Probable glutathione S-transferase	LOC107824839	GST, gst	EC:2.5.1.18
A0A1S4D3A3	Probable glutathione S-transferase	LOC107825557	GST, gst	EC:2.5.1.18
A0A1S4BG77	SEC1 family transport protein SLY1-like isoform X1	LOC107807927		
A0A1S4AX25	Vesicle-associated membrane protein 7	LOC107802228	VAMP7	
A0A1S3ZFC4	Probable methyltransferase PMT26	LOC107786067	PMT	EC:2.1.1.53

### Enzyme Activity Validation

**FIGURE 7:**
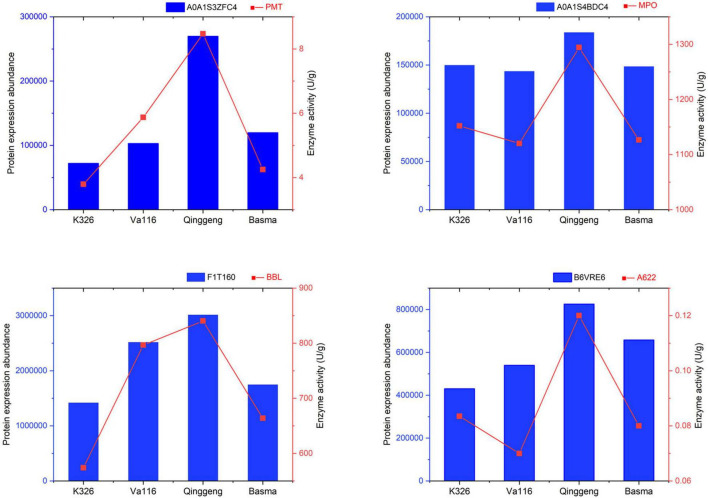
Validation of proteomics data through enzyme activity determination. A0A1S3ZFC4, A0A1S4BDC4, F1T160, and B6VRE6 are the protein accessions numbers of genes putrescine *N*-methyltransferase (PMT), *N*-methylputrescine oxidase (MPO), berberine bridge enzyme-like (BBL), and isoflavone reductase-like protein (A622), respectively.

To verify the quality of proteomics and differential expression level data, four proteins were selected for enzyme activity determination. In the nicotine synthesis pathway, PMT catalyses the conversion of putrescine into *N*-methyl putrescine, MPO catalyses the conversion of *N*-methyl putrescine into 4-methylaminobutyl ether, and A622 and BBLs combine nicotinic acid with *N*-methyl dihydropyrrole salt to synthesise nicotine. The changes in the expression of the selected proteins according to enzyme activity showed a similar expression tendency to the proteomics data (Figure 7), indicating that the proteomics profiling data were reliable.

## Discussion

Nicotine has a multi-channel utilisation value, and obtaining a high concentration of nicotine can promote the development of agricultural, pharmaceutical, and chemical industries (Yin, 2018). However, to avoid addiction to cigarette products, the nicotine content in cigarettes has been recommended to be limited to a specific range (2.5–3.5%) (Chen et al., 2004). Therefore, regulating the nicotine content in tobacco can be useful in promoting the application of nicotine in other fields. In recent years, the molecular genetics research on nicotine metabolism has been deepened, and some important genes related to nicotine synthesis, transport and transformation have been cloned successively (Hildreth et al., 2011; Bian et al., 2022), which has played an important role in promoting the research on the mechanism of nicotine synthesis and metabolism and tobacco genetics and breeding. Nicotinoids is a long-distance transport metabolite, and the whole process is also involved in synthases and transporters (Dewey and Xie, 2013). Moreover, the related intermediate metabolites are jointly controlled by adjacent metabolic pathways. Therefore, research on the mechanism of nicotine synthesis should not be limited to the nicotine synthesis pathway.

Proteomics is considered the main pillar of functional genomics, which holds great importance in the analysis of the mechanisms of trait differences between materials (Hochholdinger et al., 2018). Currently available mass spectrometers can scan in the form of DIA to achieve efficient and ready screening of biomarkers (Tsou et al., 2015; Demichev et al., 2020). Our early-stage experiment showed that DIA LC-MS is suitable for tobacco root tips for the detection and analysis of proteomics samples (Mo et al., 2021a). Therefore, in this study, we used the DIA technology to perform a comprehensive proteomics analysis of 12 samples of four tobacco varieties, and a total of 31,259 distinct peptides and 6,018 proteins were identified, which provided a material premise to further understand differences in the nicotine content and synthesis mechanisms. Further analysis showed that the differentially expressed proteins in different materials were mainly involved in the transport and metabolism of substances, and could affect the accumulation of intermediate metabolites of nicotine synthesis from different metabolic pathways.

After nicotinic synthesis, it is first necessary to cross the plasma membrane in the root to be transported to extracellular and thus transferred to the leaves for accumulation (Hashimoto and Yamada, 2003). It was found that the vesicle transport regulator, NtGEF, may indirectly affect the accumulation of nicotine content in leaves by mediating the localisation of nicotine transporter in vacuoles (Yin et al., 2018), and the results showed that vesicle transport was also involved in nicotine transport. Vesicle transport plays an indispensable role in the process of substances transport. Firstly, vesicles must fuse with target cells, and this process is promoted by a protein called soluble *N*-ethylmaleimide sensitive factor adaptor protein receptors (SNAREs) (Gao et al., 2017). Associated Sec1/Munc18 (SM) proteins in Arabidopsis were found to involve intimal transport between the ER and the Golgi apparatus (Blatt and Thiel, 2018). Vehicle associated membrane proteins 721 and 722 were involved in the transport of substances necessary for plant growth and defence response (Yi et al., 2013; Yun et al., 2013). In this study, we found that SEC1 family transport protein SLY1-like isoform X1 (A0A1S4BG77) and vesicle-associated membrane protein 711-like (A0A1S4AX25) were highly expressed in the high nicotine material Qinggeng. And Go annotation showed that they all bear the function of vacuole mediated transporter in the biological process. Therefore, we speculated that the increased nicotinic content of the Qinggeng material may be driven by these transmembrane transporters, so that the nicotine synthesised in roots can be efficiently transferred to the leaves for accumulation.

Nicotine is a nitrogen-containing compound (Yamada and Sato, 2021), and the intake and metabolism of nitrogen in plants can affect the synthesis and accumulation of nicotine (Shang et al., 2017). Previous studies have shown that over-expression of aspartate aminotransferase (AAT) genes in rice resulted in altered nitrogen metabolism (Zhou et al., 2009). In this study, we found that AAT was significantly upregulated, so we speculated that the upregulation of AAT in tobacco also improved nitrogen metabolism in plants and thus was able to synthesise more nicotinoids. The synthesis of nicotinoids involves many important metabolites, among which arginine and ornithine are the two most important precursor substances (Rubén Alcázar, 1999). Our results showed that these proteins are concentrated in tropane, piperidine and pyridine alkaloid biosynthesis, ornithine metabolism, arginine metabolism, alanine, aspartate and glutamate metabolism, glutathione metabolism, and arginine and proline metabolism. And these pathways can directly or indirectly affect the synthesis of putrescine, which provides a rich material source for the synthesis of nicotine. Putrescine *N*-methyltransferase (PMT) transferase is the largest rate limiting enzyme, which catalyses putrescine to form *N*-methylputrescine (Biastoff et al., 2009). In this study, we found that the expression of probable methyltransferase PMT26 (A0A1S3ZFC4) in Qinggeng was significantly higher than that in the other three low nicotine materials, indicating that the increase of catalytic enzyme content also played an important role in the synthesis of nicotine.

A majority of the DEPs identified in plant proteomics are usually enzymes (Lee et al., 2008). They play a vital role in many physiological, biochemical, and signalling pathways and can connect proteome with metabolome (Niu et al., 2018). Proteomics data can be explained and verified by enzyme activity determination to elucidate the mechanisms of protein (enzyme) function and enzyme-related biochemical or metabolic pathways (Boersema et al., 2015; Dhakarey et al., 2017). Xia et al. (2018) used the enzymatic reaction in combination with proteomics to elucidate the seed metabolic mechanism affected by temperature during seed dormancy and germination. In our study, the proteomics data were complemented with the enzyme activity data of four enzymes associated with nicotine synthesis, and the results indicated changes in the expression of the selected proteins. Enzyme activity showed a similar expression tendency to the proteomics data in all four varieties, which indicated that the proteomics profiling data were reliable.

In conclusion, our research explored the hub regulatory proteins of nicotine synthesis from the perspective of proteomics, and revealed that various metabolic pathways affect nicotine synthesis by regulating the formation of putrescine. The results are of great significance for regulating the nicotine content in tobacco and breeding new tobacco varieties with appropriate nicotine content. At the same time, nicotine is a secondary metabolite transported over long distances. Its synthesis and accumulation are not only controlled by its own genotype, but also affected by the growth state of roots, stems, and leaves. Therefore, in the follow-up research, we should also pay more attention to the synergy of source, sink and flow in plants to broaden the network of nicotine metabolism at the overall level.

## Data Availability Statement

The datasets presented in this study can be found in online repositories. The names of the repository/repositories and accession number(s) can be found below: The data access link in ProteomeXchange is: http://proteomecentral.proteomexchange.org/cgi/GetDataset?ID=PXD028624; The data access link in iProX is: https://www.iprox.cn/page/project.html?id=IPX00035 10000.

## Author Contributions

ZM and RL conceived and designed the experiments. ZM, LD, ZT, KP, WL, and YK performed the experiments. ZM, YP, QN, YH, and RL analysed the data and contributed to the preparation of reagents and materials, use of analytical tools, and manuscript writing. All authors contributed to the article and approved the submitted version.

## Conflict of Interest

The authors declare that the research was conducted in the absence of any commercial or financial relationships that could be construed as a potential conflict of interest.

## Publisher’s Note

All claims expressed in this article are solely those of the authors and do not necessarily represent those of their affiliated organizations, or those of the publisher, the editors and the reviewers. Any product that may be evaluated in this article, or claim that may be made by its manufacturer, is not guaranteed or endorsed by the publisher.

## Footnotes

1
https://www.blast2go.com/

2
https://www.omicshare.com/tools

3
http://www.kegg.jp/
