# Supplementary material for: Proteomics and Co-expression Network Analysis Reveal the Importance of Hub Proteins and Metabolic Pathways in Nicotine Synthesis and Accumulation in Tobacco (Nicotiana tabacum L.)
Source: Front Plant Sci. 2022 Apr 28;13:860455. doi: 10.3389/fpls.2022.860455 (PMC9096834; doi:10.3389/fpls.2022.860455)
Supplement: Supplementary file 1 [file Data_Sheet_1.docx]

**Supplementary file S1：The imformation of piptides**

**Sequence**: The amino acid sequence of peptide. Describe the composition of amino acid sequence of peptide, in which lowercase letters are modified amino acids.

**Modifications**: Describe the modification of amino acids, the location and mode of modification.

**Charge**: Charge number of peptide.

**Missed.Cleavages**: Missing cleavage of peptides by trypsin.

**PEP.IsProteinGroupSpecific**: Whether the peptide is unique to the protein cluster, true or false.

**PEP.IsProteotypic**: Whether the peptide is unique to the protein, true or false.

**PG.Cscore**: Score the identified protein group, and the higher the score, the more credible it is, usually about 1 points.

**PG.ProteinAccessions**：Protein number in the protein sequence database (FASTA database).

**PG.ProteinDescriptions**：Description of protein information.

**PG.ProteinGroups**：Proteome accession number.

**PG.ProteinNames**：Protein accession number.

**K326, Va116, Basma ,Qinggeng**: Four material names used in the study.

**-1, -2, -3**：Represents three biological replicates, respectively.

**QC**: Mixed samples of four materials.

**Supplementary file S2：Quantitative list of protein identification**

**Protein ID**: The protein ranked first in the Protein Group.

**Protein Accession**：Protein number in the protein sequence database (FASTA database).

**Protein Description**：Protein function description information ranking first in Protein Description.

**Gene Name**：The name of the gene corresponding to the protein that ranks first in the gene column in the protein group.

**PG.ProteinGroups**：Proteome accession number.

**PG.ProteinAccessions**：Protein number in the protein sequence database (FASTA database).

**PG.Genes**：Gene name.

**PG.ProteinDescriptions**：Description of protein information.

**PG.ProteinNames**：Protein accession number.

**SequenceNumber**：The number of unique peptides identified by this proteome.

**K326, Va116, Basma ,Qinggeng**: Four material names used in the study.

**-1, -2, -3**：Represents three biological replicates, respectively.

**QC**: Mixed samples of four materials

**Supplementary file S3：The proteins used for WGCNA analysis**

**Protein ID**: Protein number in the protein sequence database (FASTA database).

**Columns B to M**: Columns B to M are the expression amount of each protein in each material.
